# Relaxin Deficiency Leads to Uterine Artery Dysfunction During Pregnancy in Mice

**DOI:** 10.3389/fphys.2018.00255

**Published:** 2018-03-22

**Authors:** Sarah A. Marshall, Sevvandi N. Senadheera, Maria Jelinic, Kelly O'Sullivan, Laura J. Parry, Marianne Tare

**Affiliations:** ^1^School of BioSciences, The University of Melbourne, Parkville, VIC, Australia; ^2^Department of Physiology and Monash Rural Health, Monash University, Melbourne, VIC, Australia

**Keywords:** relaxin, pregnancy, uterine artery, myogenic tone, smooth muscle reactivity, endothelium-dependent relaxation, calcium-activated potassium channels

## Abstract

The uterine vasculature undergoes profound adaptations in response to pregnancy. Augmentation of endothelial vasodilator function and reduced smooth muscle reactivity are factors contributing to uterine artery adaptation and are critical for adequate placental perfusion. The peptide hormone relaxin has an important role in mediating the normal maternal renal vascular adaptations during pregnancy through a reduction in myogenic tone and an increase in flow-mediated vasodilation. Little is known however about the influence of endogenous relaxin on the uterine artery during pregnancy. We tested the hypothesis that relaxin deficiency increases myogenic tone and impairs endothelial vasodilator function in uterine arteries of late pregnant relaxin deficient (*Rln*^−/−^) mice. Reactivity of main uterine arteries from non-pregnant and late pregnant wild-type (*Rln*^+/+^) and *Rln*^−/−^ mice was studied using pressure and wire myography and changes in gene expression explored using PCR. Myogenic tone was indistinguishable in arteries from non-pregnant mice. In late pregnancy uterine artery myogenic tone was halved in *Rln*^+/+^ mice (*P* < 0.0001), an adaptation that failed to occur in arteries from pregnant *Rln*^−/−^ mice. The role of vasodilator prostanoids in the regulation of myogenic tone was significantly reduced in arteries of pregnant *Rln*^−/−^ mice (*P* = 0.02). Agonist-mediated endothelium-dependent vasodilation was significantly impaired in non-pregnant *Rln*^−/−^ mice. With pregnancy, differences in total endothelial vasodilator function were resolved, although there remained an underlying deficiency in the role of vasodilator prostanoids and alterations to the contributions of calcium-activated K^+^ channels. Fetuses of late pregnant *Rln*^−/−^ mice were ~10% lighter (*P* < 0.001) than those of *Rln*^+/+^ mice. In conclusion, relaxin deficiency is associated with failed suppression of uterine artery myogenic tone in pregnancy, which likely contributes to reduced uteroplacental perfusion and fetal growth restriction.

## Introduction

Normal growth and development of the fetus depends on the adequate delivery of oxygen and nutrients to the placenta, coupled with dramatic increases in maternal cardiac output, plasma volume and maternal uterine blood flow (Osol and Mandala, 2009; Conrad and Davison, 2014). Many of these maternal cardiovascular adaptations are under the influence of circulating steroid hormones and growth factors (Chang and Lubo, 2008; Hu et al., 2011; Corcoran et al., 2014). Despite increases in uterine blood flow, global uterine perfusion pressure is maintained relatively constant in pregnancy due to decreases in vascular resistance. Structural and functional adaptations of the uterine arteries during pregnancy mediate the reduction in vascular resistance in this vascular bed (Veerareddy et al., 2002; Osol and Mandala, 2009; Mandala and Osol, 2012). Failure of the uterine vasculature to properly adapt to pregnancy compromises placental perfusion, with chronic reductions in uterine blood flow linked to fetal growth restriction and death (Lang et al., 2003).

Vasoconstriction in response to an increase in intraluminal pressure, myogenic tone, is a fundamental property that regulates blood flow in many arteries (Veerareddy et al., 2002; Davis, 2012). In late gestation, myogenic tone is significantly reduced in uterine, mesenteric and renal arteries (Sherwood et al., 1980; Meyer et al., 1993; Novak et al., 2002; Veerareddy et al., 2002; Cooke and Davidge, 2003; Xiao et al., 2010). The reduction in myogenic tone of uterine arteries, together with additional functional adaptations including upregulation of endothelium-dependent vasodilation and smooth muscle refractoriness to vasoconstrictor stimuli (Ni et al., 1997; Veerareddy et al., 2002; Cooke and Davidge, 2003; Chang and Lubo, 2008; Withers et al., 2009; Hu et al., 2011), all contribute to enhanced perfusion of the utero-placental unit. Incomplete functional and structural adaptations of the uterine vasculature during pregnancy precipitates serious complications of pregnancy including fetal growth restriction, hypertension of pregnancy and pre-eclampsia (Osol and Mandala, 2009).

The peptide hormone relaxin plays an important role in mediating some of the maternal renal and systemic hemodynamic adaptations in pregnancy (Conrad and Davison, 2014). The highest circulating concentrations of relaxin occur during pregnancy with divergent levels between species. Relaxin levels peak in the last half of pregnancy in rodents (Sherwood et al., 1980) but at the end of the first trimester in humans (Stewart et al., 1990). Correlated with this increase in relaxin in pregnant women are reductions in uterine and renal artery resistance (Smith et al., 2006). Importantly, women with ovarian failure who conceive with donor eggs, *in vitro* fertilization, or embryo transfer (with no measureable circulating relaxin) fail to undergo systemic vasodilation during pregnancy and are at an increased risk of developing adverse outcomes of pregnancy (Conrad and Davison, 2014).

Few studies have explored the effects of endogenous relaxin on the uterine vasculature during pregnancy. Relaxin receptors (RXFP1) are detected in mouse and rat renal arteries, aorta (Novak et al., 2006; Ferreira et al., 2009; Jelinic et al., 2014) and uterine arteries (Vodstrcil et al., 2012) of pregnant mice and rats. Monoclonal antibody (MCA1) administration to neutralize circulating relaxin in pregnant rats attenuated renal and systemic vasodilation (Novak et al., 2001), and increased uterine artery passive circumferential wall stiffness (Vodstrcil et al., 2012). This was the first evidence that relaxin deficiency could impact upon vascular function in pregnancy. Later studies in relaxin-deficient (*Rln*^−/−^) mice demonstrated impaired mesenteric artery reactivity (Marshall et al., 2016, 2017a) and impaired uterine artery remodeling (Gooi et al., 2013) associated with stiffer uterine arteries and reduced fetal weights. In this study we tested the hypothesis that relaxin deficiency in pregnancy impairs the normal adaptation of uterine artery function, thereby affecting fetal growth. The aims of this study were to investigate if relaxin deficiency: (i) increased uterine artery myogenic tone in pregnancy, and (ii) impaired agonist-induced endothelium-dependent vasodilation. A multi-gene quantitative PCR Array was also used to investigate the signaling molecules underlying altered mechanisms of uterine artery function.

## Materials and methods

### Animals

All animal experiments were approved by The University of Melbourne Animal Experimental Ethics Committee (AECC 1212387) and conducted in accordance within the Australian Code of Practice and the National Health and Medical Research Council guidelines. This study used the *Rln*^−/−^ mouse backcrossed on a C57BL/6J background to the F_14_ generation and wild-type (*Rln*^+/+^) littermates of the same strain (Zhao et al., 1999). Mice were housed at The University of Melbourne animal house facility located in the School of BioSciences, on a 12 h light: 12 h dark cycle at 20°C, with access to standard food pellets (Barastock, Pakenham, Vic, Australia) and water *ad libitum*. Genotypes of mice were confirmed by RT-PCR analysis of ear clips, as previously described (Zhao et al., 1999). Age matched (3–5 months old) *Rln*^+/+^ and *Rln*^−/−^ mice were studied in two cohorts, non-pregnant (estrus) and late pregnant (day 17.5 of pregnancy).

### Sample collection and uterine artery isolation

On the day of the experiment, mice were weighed, anesthetized with 2% isofluorane and euthanized by cervical dislocation. Uterine arteries were isolated into ice-cold Krebs physiological solution with HEPES (PSS-HEPES) containing (mM): NaCl 112, NaHCO_3_ 25, KCl 4.7, MgSO_4_ 1.2, KH_2_PO_4_ 0.7, HEPES 10, D-glucose 11.6, and CaCl_2_ 2.5 (pH 7.4). Main uterine arteries were carefully cleaned of loose connective and adipose tissue. The right uterine artery was used for pressure myography and the left for wire myography. Remaining segments of arteries were snap frozen in liquid nitrogen and stored at −80°C for later analysis.

### Pressure myography

Leak-free segments of uterine artery were cannulated on glass micropipettes of a pressure myograph (Living Systems Instrumentation, Burlington, VT, USA) and outside diameter measured using video microscopy (Diamtrak software, Adelaide, SA, Australia). Experiments were performed in the absence of intraluminal flow, with continuous superfusion (4 ml/min) with PSS-HEPES at 37°C. At the start of each experiment arteries were acclimatized for 40 min at 50 mmHg. Smooth muscle and endothelial cell viability was then tested, as previously described (Tare et al., 2011). Myogenic tone development was assessed over the intraluminal pressure range of 10–120 mmHg (10, 30, 50, 60, 80, 100, 120 mmHg) with pressure increments every 10 min. This was repeated following 30 min of pre-incubation with N^ω^-nitro-l-arginine methyl ester (L-NAME; 200 μmol/L) and L-NAME and indomethacin (Indo; 1 μmol/L) to investigate the contribution of nitric oxide (NO) and prostanoids, respectively, in the modulation of myogenic tone. In order to determine the arterial passive diameters at each pressure, at the conclusion of each experiment the pressurization protocol was repeated in a 0 mmol/L calcium (Ca^2+^)-containing PSS-HEPES and 2mM EGTA (after 30 min of pre-incubation in the buffer).

### Wire myography

Agonist-induced vascular reactivity was assessed, as previously described (Leo et al., 2014a,b). Briefly, main uterine arteries, ~2 mm in length were mounted on a four-channel wire myograph (Danish Myo Technology, Aarhus, Denmark). To examine alpha-adrenoceptor mediated contraction, arteries were exposed to increasing concentrations of the α_1_-adrenoceptor agonist, phenylephrine (PE, 1–0.1 mmol/L), applied cumulatively. Contractions were expressed as a percentage of the contraction evoked by 100 mM high potassium physiological saline solution (KPSS, isotonic replacement of Na^+^ with K^+^). To assess endothelium-dependent and –independent vasodilator function, uterine arteries were precontracted to a similar level (60–70% of maximum KPSS contraction) using PE (0.1–3 μmol/L), and concentration-response curves to the endothelium-dependent agonists acetylcholine (ACh, 0.1 nmol/L to 10 μmol/L), or bradykinin (BK, 0.1 nmol/L to 1 μmol/L), and the endothelium-independent agonists sodium nitroprusside (SNP, 0. 1 nmol/L to 10 μmol/L) and iloprost (0.1 nmol/L to 1 μmol/L) were determined (Marshall et al., 2016). Relaxation was expressed as a percentage of the level of preconstriction. Responses to ACh and BK were also examined after 30 min of incubation with different combinations of pharmacological blockers, including L-NAME and Indo. The residual relaxation after blockade of nitric oxide synthase (NOS) and cyclooxygenase (COX) is attributed to endothelium-derived hyperpolarization (EDH). The contribution of intermediate- and small-conductance calcium activated potassium channels to EDH-mediated relaxation was assessed by pre-incubation with TRAM-34 (5 μmol/L) and apamin (0.1 μmol/L), respectively, in the presence of L-NAME + Indo.

### Quantitative PCR array

Frozen uterine arteries from pregnant *Rln*^+/+^ (*n* = 5) and *Rln*^−/−^ (*n* = 5) mice were placed in pre-chilled Wig-L-Bug® capsules and pulverized in a Digital Wig-L-Bug® amalgamator (Dentsply-Rinn, Elgin, IL, USA). Pulverized tissues were resuspended in 1 ml TriReagent (Ambion Inc., Scoresbury, VIC, Australia) and total RNA was then extracted as described previously with an extra final wash in 70% ethanol (Vodstrcil et al., 2012; Leo et al., 2014b). RNA pellets were resuspended in 12 μl RNA Secure™ (Ambion). Quantity of RNA was analyzed using the NanoDrop® ND100 Spectrophotometer (Thermo Fischer Scientific Australia Pty Ltd, Scoresby, VIC, Australia) with A_260_:A_280_ ratios > 1.8 indicating sufficient quality for qPCR analysis. On average, the accumulated uterine artery of 4 pregnant mice (after functional studies) yielded ~0.7 μg of RNA to create 1 data point, leaving insufficient RNA to verify integrity via gel electrophoresis. First strand cDNA synthesis was performed using the RT^2^ First Strand Kit (QIAGEN, Chadstone, VIC, Australia), as per kit instructions using 0.5 μg of total RNA per reaction. The qPCR was performed using RT^2^ Profiler^TM^ PCR Hypertension Array for Mouse analyzing 84 genes (QIAGEN; Cat. No. PARN-037Z) as per kit instructions for the AB Applied Biosystems ViiA7 PCR machine (Life Technologies, Mulgrave, VIC, Australia) in 20 μl volume fast reactions. β-actin (*Actb*), β-glucuronidase (*Gusb*), and heat shock protein 90-α-B1 (*Hsp90ab1*) were the reference genes selected by the manufacturer. For each gene, the mean Ct-value for the reference genes was subtracted from the mean gene of interest Ct-value to normalize gene of interest to the reference genes. The fold difference and statistical differences in expression between *Rln*^+/+^ and *Rln*^−/−^ mice for each gene were calculated using the QIAGEN GeneGlobe Data Analysis Centre using the 2^−Δ*Ct*^ method of analysis.

### Fetal frequency distribution curve and placental weights

Fetuses were harvested with laparotomy, blotted and wet weights were measured (number of fetuses, *Rln*^+/+^ = 161 from 21 L and *Rln*^−/−^ = 150 from 17 L). Histograms were constructed with the measurements and the 5th percentile weight was calculated, as described previously (Dilworth et al., 2011). After detaching the placentae, fetal membranes were removed, and the placentae dried on tissues before weighing (number of placentas, *Rln*^+/+^ = 36 from 7 L and *Rln*^−/−^ = 40 from 8 L).

### Chemicals

All drugs were purchased from Sigma-Aldrich. Drugs were dissolved in distilled water, with the exception of indomethacin (0.1 mol/L sodium carbonate) and TRAM-34 (dimethyl sulfoxide), with subsequent dilutions in distilled water.

### Calculations and statistical analysis

All results are expressed as mean ± SEM; “n” represents the number animals per group, except in gene analysis where each “n” represents pooled main uterine arteries from *n* = 3–5 animals. Myogenic tone was calculated as ((D_1_ – D_2_)/D_1_) × 100, where D_1_ is the outer diameter in Ca^2+^ free PSS and D_2_ is the outer diameter in the presence of extracellular Ca^2+^. The relative contribution of NO and vasodilator prostanoids in the regulation of myogenic tone was determined by analyzing the area under the curve (AUC). In brief, the role of the vasodilator prostanoid component was calculated by subtracting AUC in the presence of L-NAME+Indo from that obtained in L-NAME alone. Similarly, the component of the response mediated by NO was determined by subtracting the AUC in L-NAME from AUC obtained in the absence of inhibitors.

For the wire myography experiments, sigmoid curves were fitted to agonist-induced concentration response data using the least squares method (Prism version 6.0, GraphPad Software, San Diego, CA, USA) to calculate the sensitivity of each agonist (pEC_50_). Maximum relaxation (R_max_) to ACh, BK, SNP and iloprost was measured as a percentage of pre-constriction to PE. The relative contribution of NO, vasodilator prostanoids and EDH to relaxation evoked by ACh or BK was determined by analyzing the AUC of the ACh or BK response curves, as described previously (Marshall et al., 2017b). The intermediate-conductance Ca^2+^-activated potassium channel (IK_Ca_) contribution to EDH-mediated relaxation was determined by subtracting the AUC in L-NAME+Indo+TRAM-34 from that obtained with L-NAME+Indo. Similarly, small-conductance Ca^2+^-activated potassium channel (SK_Ca_) contribution to EDH-mediated relaxation was determined by subtracting AUC in L-NAME+Indo+TRAM-34+apamin from that in L-NAME+Indo+TRAM-34. Group pEC_50_, R_max_, and AUC values were compared using 1-way ANOVA with Bonferroni *post-hoc* analysis or Student's independent *t*-tests. Concentration-response curves were also analyzed via two-way ANOVA with Bonferroni *post-hoc* analysis (treatment vs. concentration). *P* < 0.05 was considered statistically significant. The estimate of marginal means of fetal and placental weights, adjusted for dams and litter size, was analyzed using SPSS (version 25.0, SPSS, Chicago, IL, USA).

## Results

### Influence of relaxin deficiency on myogenic tone development

#### Relaxin deficiency does not affect myogenic tone development in uterine arteries of non-pregnant mice

**Figure 1:**
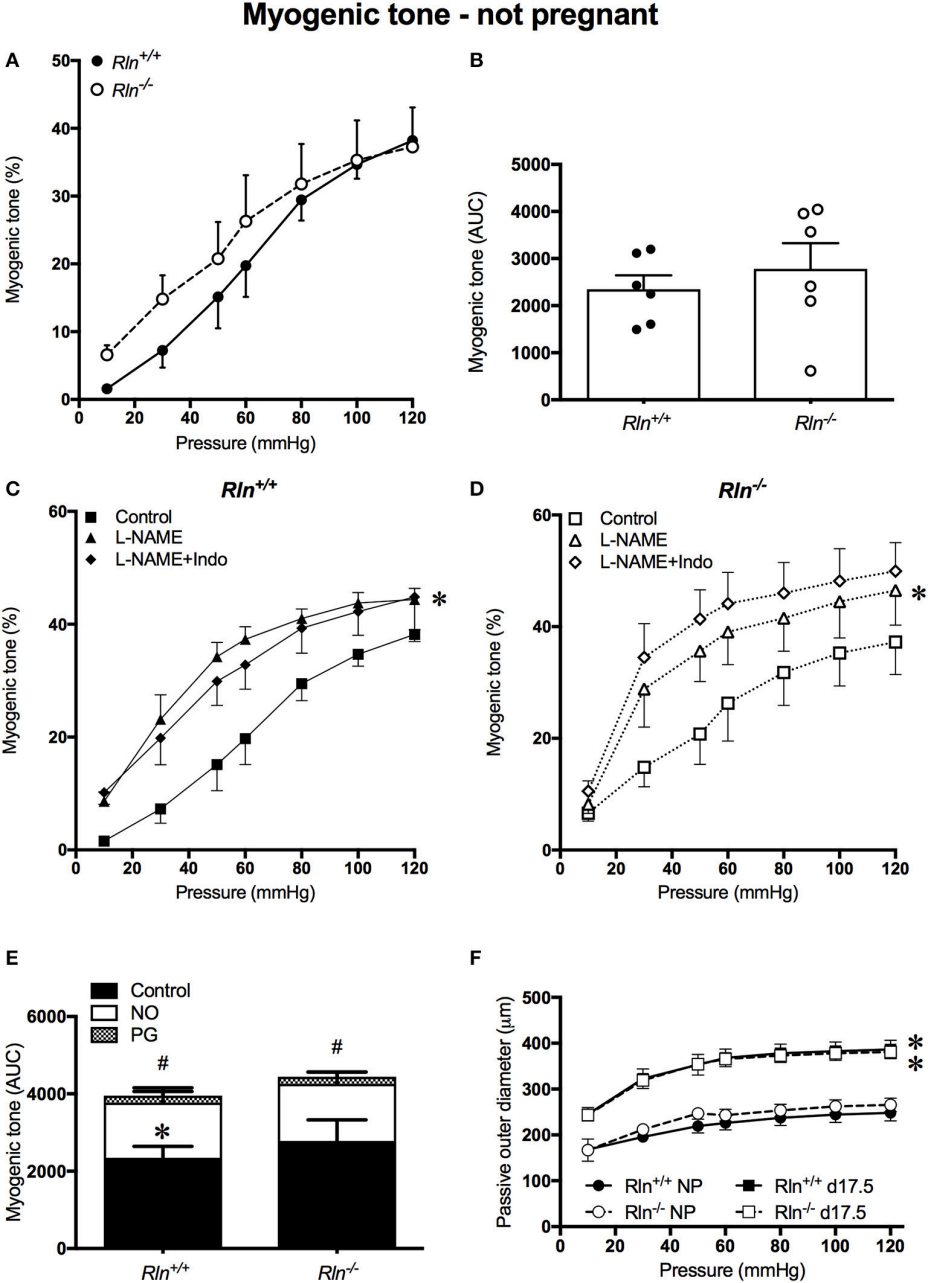
Myogenic tone development in the main uterine artery of non-pregnant *Rln*^+/+^ (filled black) and *Rln*^−/−^ mice (filled white) (*n* = 5 per group). **(A)** Myogenic tone development with increasing pressure (mmHg) and **(B)** area under the curve (AUC). Myogenic tone of the main uterine artery from non-pregnant **(C)** *Rln*^+/+^ and **(D)** *Rln*^−/−^ mice after pre-treatment of arteries without (square; control) or with the NOS inhibitor (triangle) L-NAME or (diamond) L-NAME and the COX inhibitor Indo (L-NAME+Indo). **(E)** AUC analysis of relative contributions of NO and vasodilator prostanoids (PG) to myogenic tone development in uterine arteries from non-pregnant *Rln*^+/+^ and *Rln*^−/−^ mice. **(F)** Passive outer diameters of the main uterine arteries after intraluminal pressurization (10–120 mmHg) in *Rln*^+/+^ and *Rln*^−/−^ mice in estrus (NP: circles) and in late pregnancy (d17.5; squares) (*n* = 6–8 per group). ^*^P < 0.05 two-way ANOVA control vs. L-NAME **(C,D)** or not pregnant vs. pregnant **(F)**; ^#^*P* < 0.05 one-way ANOVA control vs. PG; ^*^P < 0.05 one-way ANOVA NO vs. PG **(E)**.

Uterine arteries from non-pregnant *Rln*^+/+^ and *Rln*^−/−^ mice developed myogenic tone with intraluminal pressurization, in the presence of Ca^2+^ (Figure 1A). The magnitude of myogenic tone development was not significantly different between uterine arteries of non-pregnant *Rln*^+/+^ and *Rln*^−/−^ animals (Figures 1A,B). Endothelium-derived vasodilators can modulate myogenic tone development. Here, we examined the contributions of NO and vasodilator prostanoids in this role. Following inhibition of NOS activity with L-NAME, myogenic tone development was significantly increased in uterine arteries from *Rln*^+/+^ (*P* = 0.02) and *Rln*^−/−^ (*P* = 0.02) mice (Figures 1C,D). Subsequent inhibition of vasodilator prostanoid synthesis with Indo in the presence of L-NAME was without further significant effect (Figures 1C,D). AUC analyses revealed that the overall magnitude of increase in tone in the presence of NOS and COX inhibitions was not different between *Rln*^+/+^ and *Rln*^−/−^ mice (Figure 1E). In non-pregnant mice vasodilator prostanoids have a negligible role in the modulation of myogenic tone in the uterine artery. In the absence of Ca^2+^, no differences were found between the passive outer diameter of uterine arteries of *Rln*^+/+^ and *Rln*^−/−^ mice. With pregnancy there was a significant increase is passive outer diameter of the uterine artery (*P* < 0.0001; Figure 1F), with no differences between genotypes.

#### Myogenic tone development in uterine arteries is increased in pregnant relaxin-deficient mice

**Figure 2:**
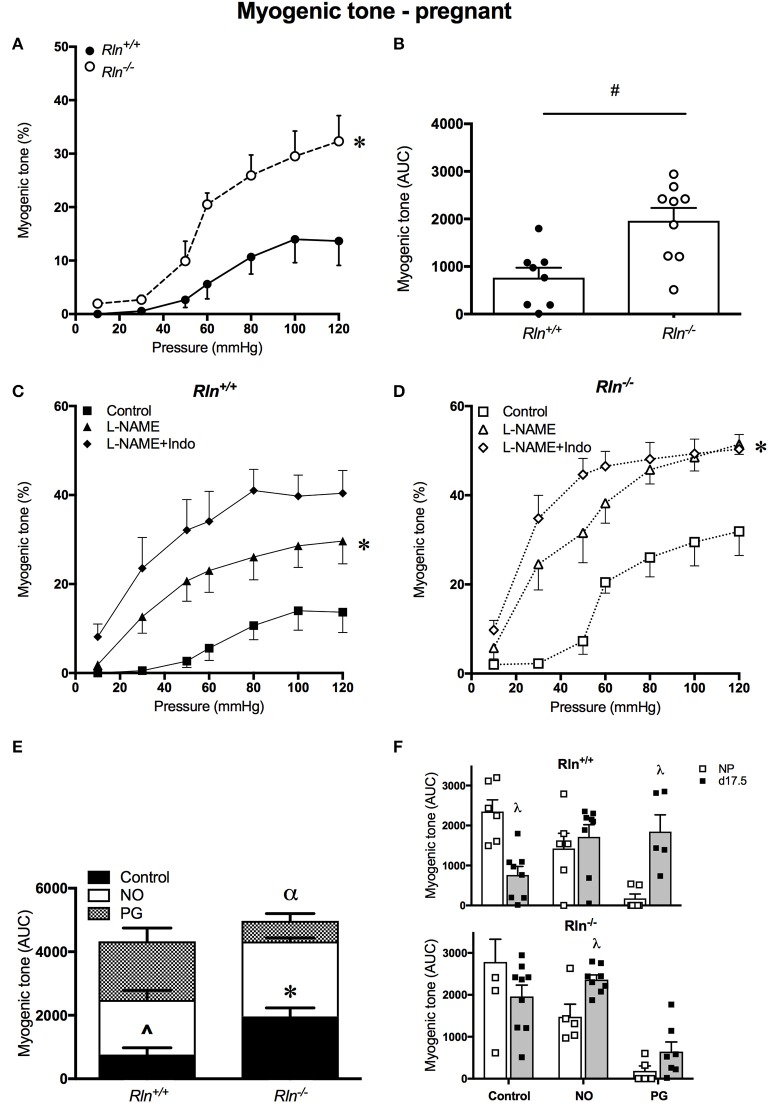
Myogenic tone development in uterine arteries of late-pregnant *Rln*^+/+^ and *Rln*^−/−^ mice. Myogenic tone: **(A)** with increasing pressure (mmHg) and **(B)** area under the curve (AUC). (*n* = 8–9 per group). Myogenic tone: late-pregnant **(C)** *Rln*^+/+^ and **(D)** *Rln*^−/−^ mice after pre-treatment of arteries without (squares) or with the NOS inhibitor (triangle) L-NAME or (diamonds) L-NAME and the COX inhibitor Indo (L-NAME+Indo). Contribution of NO and vasodilator prostanoids (PG) to myogenic tone analyzed as AUC (*n* = 5–9 per group) in the main uterine artery from pregnant **(E)**, and compared between **(F)** non-pregnant (NP) and pregnant (d17.5) *Rln*^+/+^ and *Rln*^−/−^ mice. Data are shown as mean ± SEM. ^*^P < 0.05 compared with genotype, two-way ANOVA **(A)**, ^#^*P* < 0.05 between genotypes, Student *t*-test, ^*^P < 0.05 compared with control, two-way ANOVA control vs. L-NAME **(C,D)**; ^∧^*P* < 0.05 one-way ANOVA control vs. NO, ^*^P < 0.05 one-way ANOVA control vs. NO **(E)**; ^α^*P* < 0.05 one-way ANOVA NO vs. PG **(E)**. ^λ^*P* < 0.05 two-way ANOVA with factors pregnancy status or endothelial factors **(F)**.

In late pregnancy, myogenic tone development still occurred in uterine arteries of pregnant *Rln*^+/+^ and *Rln*^−/−^ mice. Myogenic tone was significantly blunted in arteries from pregnant *Rln*^+/+^ mice (Figure 2A, *P* < 0.0001). AUC analyses revealed that overall myogenic tone development in uterine arteries from *Rln*^−/−^ mice was double that in those of *Rln*^+/+^ mice (*P* < 0.01, Figure 2B).

Treatment with L-NAME alone significantly increased myogenic tone development in arteries of both *Rln*^+/+^ (*P* = 0.04) and *Rln*^−/−^ mice (*P* = 0.0005, Figures 2C,D). Inhibition of prostanoid synthesis had no further significant effect. AUC analysis revealed that both NO (*P* = 0.007) and prostanoids (*P* = 0.002) make important and significant contributions to the regulation of myogenic tone in the normal pregnant *Rln*^+/+^ mouse (Figure 2E). For uterine arteries from *Rln*^−/−^ mice, AUC analysis revealed that although both NO and vasodilator prostanoids contribute to the regulation of myogenic tone, the role of NO was significantly greater (*P* < 0.0001; Figure 2E).

When comparing overall AUC of myogenic tone in the uterine arteries of non-pregnant and pregnant mice, it is evident that although the NO component is upregulated in the *Rln*^−/−^ mice, there is no augmentation of the prostanoid component in these mice during pregnancy. Upregulation of the prostanoid component was marked in uterine arteries of *Rln*^+/+^ in pregnancy (Figure 2F).

### Agonist-induced endothelium-dependent relaxation

#### Endothelial dysfunction in uterine arteries of non-pregnant relaxin deficient mice

**Figure 3:**
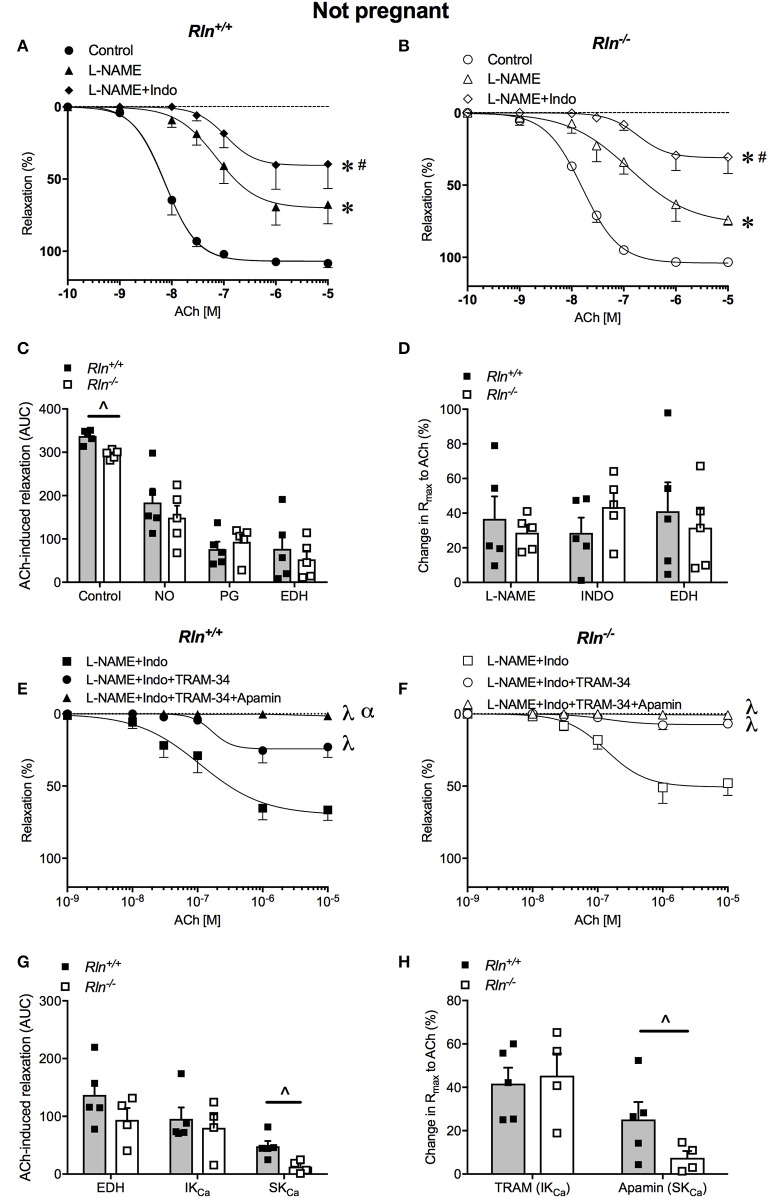
Concentration-response curves for the endothelium-dependent agonist acetylcholine (ACh) in main uterine arteries from non-pregnant **(A)** *Rln*^+/+^ and **(B)** *Rln*^−/−^ mice in the absence (circle; control) or presence of the NOS inhibitor (triangle) L-NAME or (diamond) L-NAME and the COX inhibitor Indo (L-NAME+Indo). **(C)** Area under the curve (AUC) analysis of the contribution of nitric oxide (NO), vasodilator prostanoids (PG) and endothelium-derived hyperpolarization (EDH) to ACh-induced relaxation in main uterine arteries from non-pregnant *Rln*^+/+^ and *Rln*^−/−^ mice and **(D)** the change in maximum endothelium–dependent relaxation (R_max_) after incubation with L-NAME or L-NAME+Indo; with the remaining relaxation attributed to EDH. Concentration-response curves to ACh from non-pregnant **(E)** *Rln*^+/+^ and **(F)** *Rln*^−/−^ mice after pre-treatment of arteries with (square) L-NAME+Indo, (circle) L-NAME+Indo+TRAM-34, (triangle) L-NAME+Indo+TRAM-34+apamin. **(G)** AUC analysis of EDH-mediated relaxation and the contributions of intermediate- (IK_Ca_) and small-conductance (SK_Ca_) Ca^2+^-activated potassium channels to ACh-evoked relaxation in uterine arteries of non-pregnant *Rln*^+/+^ and *Rln*^−/−^ mice and **(H)** the change in maximum relaxation (R_max_) (*n* = 5–8 per group). ^*^P < 0.0001 two-way ANOVA compared to control; ^#^*P* < 0.05 two-way ANOVA L-NAME vs. L-NAME+Indo; ^λ^*P* < 0.05 two-way ANOVA relative to L-NAME+Indo; ^α^*P* < 0.05 two-way ANOVA L-NAME+Indo+TRAM-34 vs. L-NAME+Indo+TRAM-34+apamin; ^∧^*P* < 0.05 based on genotype one-way ANOVA.

ACh induced concentration-dependent relaxation in arteries from both *Rln*^+/+^ and *Rln*^−/−^ mice (Figures 3A,B). Sensitivity to ACh was reduced by two-fold (pEC_50_, *P* < 0.05) and overall AUC for relaxation was significantly reduced in non-pregnant *Rln*^−/−^ compared with *Rln*^+/+^ mice (Figure 3C; Table 1). Maximum relaxation was not different between arteries from *Rln*^+/+^ and *Rln*^−/−^ mice (Table 1).

**Table 1:** Uterine artery reactivity in *Rln*^+/+^ and *Rln*^−^^/–^ mice.

	***Rln***^**+/+**^			***Rln***^**−/−**^		
	**pEC_50_**	**R_max_ (%)**	***n***	**pEC_50_**	**R_max_ (%)**	***n***
**NOT-PREGNANT**						
ACh – Control	8.1 ± 0.1	106.5 ± 2.3	5	7.8 ± 0.0^*^	104.0 ± 2.0	5
+L-NAME	7.1 ± 0.1^#^	69.8 ± 12.7^#^	5	7.1 ± 0.2^#^	75.2 ± 3.7^#^	5
+L-NAME+Indo	7.0 ± 0.1^#^	41.2 ± 16.7^#^	5	6.7 ± 0.1^*^^#^	36.1 ± 10.9^#^	5
ACh + L-NAME+Indo	7.0 ± 0.2	68.3 ± 7.7	5	6.9 ± 0.2	53.6 ± 8.6	4
+L-NAME+Indo+TRAM	6.8 ± 1.1	26.6 ± 8.0^‡^	5	6.9 ± 0.4	8.2 ± 2.8^‡^	4
+L-NAME+Indo+TRAM+Apa	ND	1.4 ± 1.0^‡^^§^	5	ND	0.7 ± 0.7^‡^^§^	4
BK – Control	7.3 ± 0.2	29.3 ± 16.8	5	6.7 ± 0.4	4.4 ± 1.1	5
+L-NAME	ND	0.0 ± 0.0	5	ND	0.0 ± 0.0	5
+L-NAME+Indo	ND	0.0 ± 0.0	5	ND	0.0 ± 0.0	5
SNP	7.1 ± 0.1	85.2 ± 3.7	5	7.0 ± 0.2	76.7 ± 5.0	5
Iloprost	5.9 ± 0.0	34.7 ± 5.6	5	5.8 ± 0.1	36.2 ± 2.0	4
**PREGNANT**						
ACh – Control	8.3 ± 0.1	105.3 ± 1.5	9	8.3 ± 0.3	103.7 ± 1.3	7
+L-NAME	7.3 ± 0.2^#^	92.5 ± 1.8^#^	8	7.0 ± 0.1^#^	85.6 ± 4.1^#^	7
+L-NAME+Indo	7.2 ± 0.1^#^	76.4 ± 3.8^#^	8	7.0 ± 0.1^#^	76.8 ± 6.7^#^	7
ACh +L-NAME+Indo	7.2 ± 0.1	86.7 ± 3.5	4	7.4 ± 0.1	88.3 ± 3.0	7
+L-NAME+Indo+TRAM	6.4 ± 0.1^‡^	73.7 ± 2.8^‡^	7	6.5 ± 0.1^‡^	50 ± 8.5^*^^‡^	7
+L-NAME+Indo+TRAM+Apa	5.9 ± 0.3^‡^	9.6 ± 2.6^‡^^§^	6	5.3 ± 0.2^‡^^§^	11.1 ± 5.2^‡^^§^	7
BK - Control	6.6 ± 0.3	37.5 ± 10.0	9	6.7 ± 0.3	46.4 ± 12.5	7
+L-NAME	5.6 ± 0.2^#^	11.4 ± 4.6^#^	8	6.0 ± 0.4	19.2 ± 10.2	7
+L-NAME+Indo	ND^#^^†^	0.2 ± 0.2^#^^†^	8	ND	1.08 ± 9.4^#^	7
SNP	8.9 ± 1.4	102.1 ± 1.3^+^	8	7.5 ± 0.1^+^	101.2 ± 3.0^+^	7
Iloprost	6.5 ± 0.5	55.5 ± 9.5	8	7.2 ± 0.2	38.8 ± 8.0	7

*All data presented as mean ± SEM. ND, not determined. TRAM, TRAM-34; Apa, apamin*.

* P < 0.05 compared to Rln^+/+.^

# *P < 0.05 compared to Control*.

† *P < 0.05 compared to L-NAME*.

‡ *P < 0.05 compared to L-NAME+Indo*.

§ *P < 0.05 compared to L-NAME+Indo+TRAM-34*.

+ *P < 0.05 compared with SNP non-pregnant*.

NOS inhibition significantly reduced ACh induced relaxation (R_max_ and AUC, *P* < 0.05) and sensitivity (*P* < 0.05) in uterine arteries from non-pregnant mice (Figures 3A–D; Table 1). Sensitivity and maximal response to ACh was not different between *Rln*^−/−^ and *Rln*^+/+^ mice in L-NAME (Figures 3A,B,D; Table 1). Blockade of prostanoid production caused further inhibition of relaxation. In the presence of Indo, sensitivity to ACh was lower (two-fold, *P* = 0.02) in *Rln*^−/−^ mice (Table 1). Relaxation remaining in the presence of L-NAME+Indo is attributed to EDH.

Overall AUC for relaxation was significantly smaller in arteries from *Rln*^−/−^ mice (Figure 3C). AUC analysis revealed that the contribution of NO, vasodilator prostanoids and EDH to endothelium-dependent relaxation was not different between *Rln*^+/+^ and *Rln*^−/−^ mice. The change in maximal relaxation induced by L-NAME and Indo, and the maximal relaxation due to EDH was not significantly different between groups (Figure 3D).

The relative contributions of IK_Ca_ and SK_Ca_ channels to EDH-mediated relaxation was examined in the presence of L-NAME+Indo with TRAM-34 only or a combination of TRAM-34+apamin (Figures 3E,F). Inhibition of IK_Ca_ activity with TRAM-34 did not alter sensitivity to ACh but it significantly reduced maximum EDH-mediated relaxation in arteries from both *Rln*^+/+^ (*P* = 0.006) and *Rln*^−/−^ (*P* = 0.002) mice, however the extent of reduction produced by TRAM-34 was not different between genotypes (Figures 3E–H; Table 1). Subsequent blockade of SK_Ca_ channel activity with apamin further reduced EDH-mediated relaxation (pEC_50_, R_max_, and AUC) (Figures 3E–H; Table 1). Maximum relaxation was significantly reduced in both *Rln*^+/+^ (*P* = 0.01) and *Rln*^−/−^ (*P* = 0.04) mice (Figure 3F). The effect of SK_Ca_ inhibition on maximal EDH-mediated relaxation was greater in uterine arteries from *Rln*^+/+^ compared with *Rln*^−/−^ mice (Figure 3H). EDH relaxation was all but abolished in the presence of IK_Ca_ and SK_Ca_ channel inhibition in uterine arteries from both *Rln*^+/+^ and *Rln*^−/−^ mice.

AUC analysis revealed that overall EDH relaxation was not different between *Rln*^+/+^ and *Rln*^−/−^ mice (Figure 3G). The underlying contribution of IK_Ca_ channels was unchanged but the contribution of SK_Ca_ channels was significantly (*P* < 0.05) reduced in arteries from *Rln*^−/−^ mice (Figures 3G,H).

### Influence of pregnancy on endothelium-dependent relaxation

**Figure 4:**
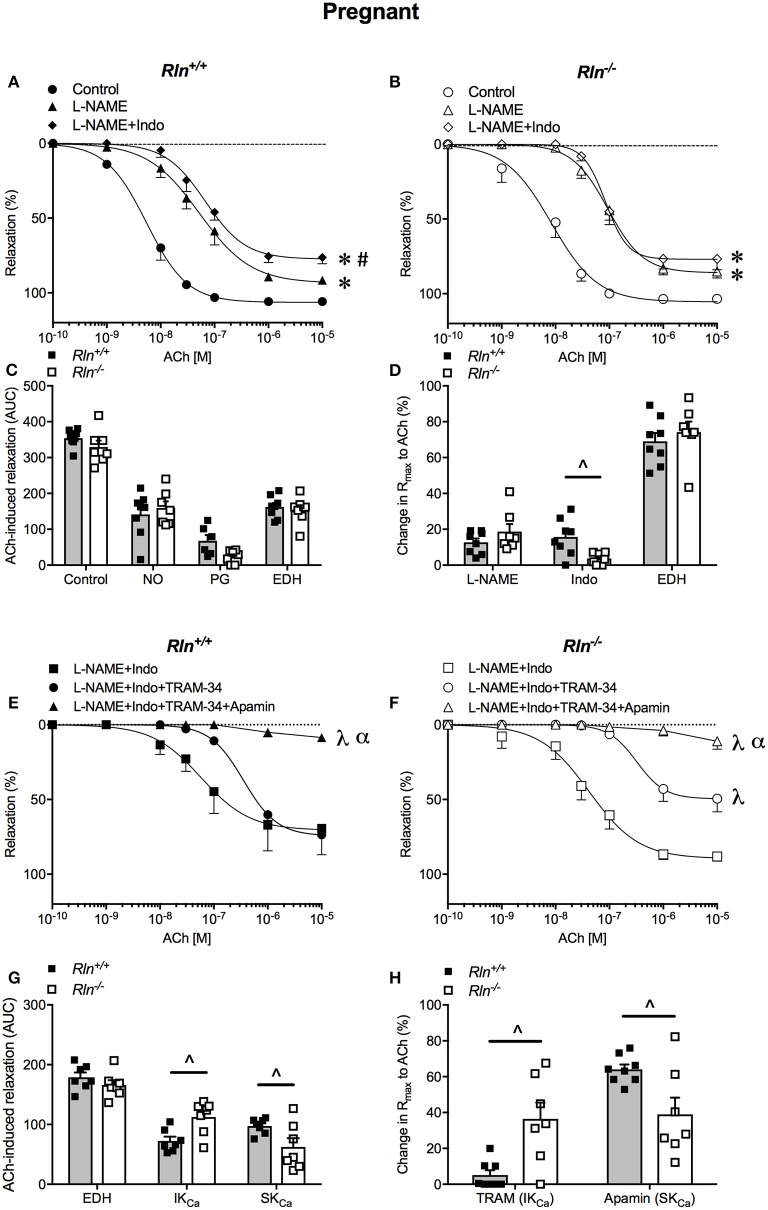
Concentration-response curves to the endothelium-dependent agonist acetylcholine (ACh) in main uterine arteries from late-pregnant **(A)** *Rln*^+/+^ and **(B)** *Rln*^−/−^ mice after pre-treatment of arteries without (circle; control) or with the NOS inhibitor (triangle) L-NAME or (diamond) L-NAME and the COX inhibitor Indo (L-NAME+Indo). **(C)** Contribution of nitric oxide (NO), vasodilator prostanoids (PG) and endothelium-derived hyperpolarization (EDH) to ACh-induced relaxation from late-pregnant *Rln*^+/+^ and *Rln*^−/−^ mice) and **(D)** change in maximum relaxation (R_max_) to ACh after incubation with L-NAME and L-NAME +Indo, with the remaining relaxation attributed to EDH. Concentration-response curves to ACh in main uterine arteries from late-pregnant **(E)** *Rln*^+/+^ and **(F)** *Rln*^−/−^ mice after pre-treatment of arteries with (square) L-NAME+Indo, (circle) L-NAME+Indo+TRAM-34, (triangle) L-NAME+Indo+TRAM-34+apamin. **(G)** Contribution of EDH, intermediate-conductance (IK_Ca_) and small- conductance (SK_Ca_) Ca^2+^-activated potassium channels to ACh-induced relaxation in main uterine arteries from non-pregnant *Rln*^+/+^ and *Rln*^−/−^ mice as analyzed as AUC and **(H)** the change in maximum relaxation (R_max_) to ACh after incubation with TRAM-34 and TRAM-34+apamin (*n* = 7–9 per group). ^*^P < 0.0001 two-way ANOVA compared with control; ^#^*P* < 0.05 two-way ANOVA L-NAME vs. L-NAME+Indo; ^λ^*P* < 0.0001 two-way ANOVA relative to L-NAME+Indo; ^α^*P* < 0.05 two-way ANOVA L-NAME+Indo+TRAM-34 vs. L-NAME+Indo+TRAM-34+apamin; ^∧^*P* < 0.05 based on genotype one-way ANOVA.

In pregnancy, there was no significant difference in uterine artery sensitivity, AUC or maximum relaxation to ACh between pregnant *Rln*^−/−^ and *Rln*^+/+^ mice (Figures 4A–C; Table 1). From the AUC analysis, the contribution of NO, vasodilator prostanoids and EDH to overall endothelium-dependent relaxation was not different between genotypes. The overall contribution of EDH to endothelium-dependent relaxation in the uterine artery was significantly augmented in pregnancy in both *Rln*^+/+^ (*P* = 0.0008) and *Rln*^−/−^ (*P* = 0.002) mice (Figure 4C vs. Figure 3C; Table 1).

Pre-incubation with L-NAME significantly reduced ACh-induced relaxation (pEC_50_, R_max_, and AUC, *P* < 0.05) in uterine arteries of pregnant mice. The effect of L-NAME on relaxation was not different between arteries from *Rln*^−/−^ and *Rln*^+/+^ mice (Figures 4A–D; Table 1). Subsequent exposure to Indo was without further significant effect on sensitivity to ACh, but maximum relaxation was further reduced in arteries from *Rln*^+/+^ (*P* = 0.018) but not *Rln*^−/−^ mice (Figure 4D).

In pregnancy, sensitivity of ACh-evoked EDH-mediated relaxation was not different between *Rln*^+/+^ and *Rln*^−/−^ mice (Figures 4E,F; Table 1). The underlying contribution of IK_Ca_ channels to EDH-mediated relaxation was significantly (*P* < 0.05) augmented, however the role of SK_Ca_ channels was significantly (*P* < 0.05) reduced in *Rln*^−/−^ mice (Figures 4E–H; Table 1).

### Impairment of BK-mediated endothelium-dependent relaxation in non-pregnant relaxin deficient mice

**Figure 5:**
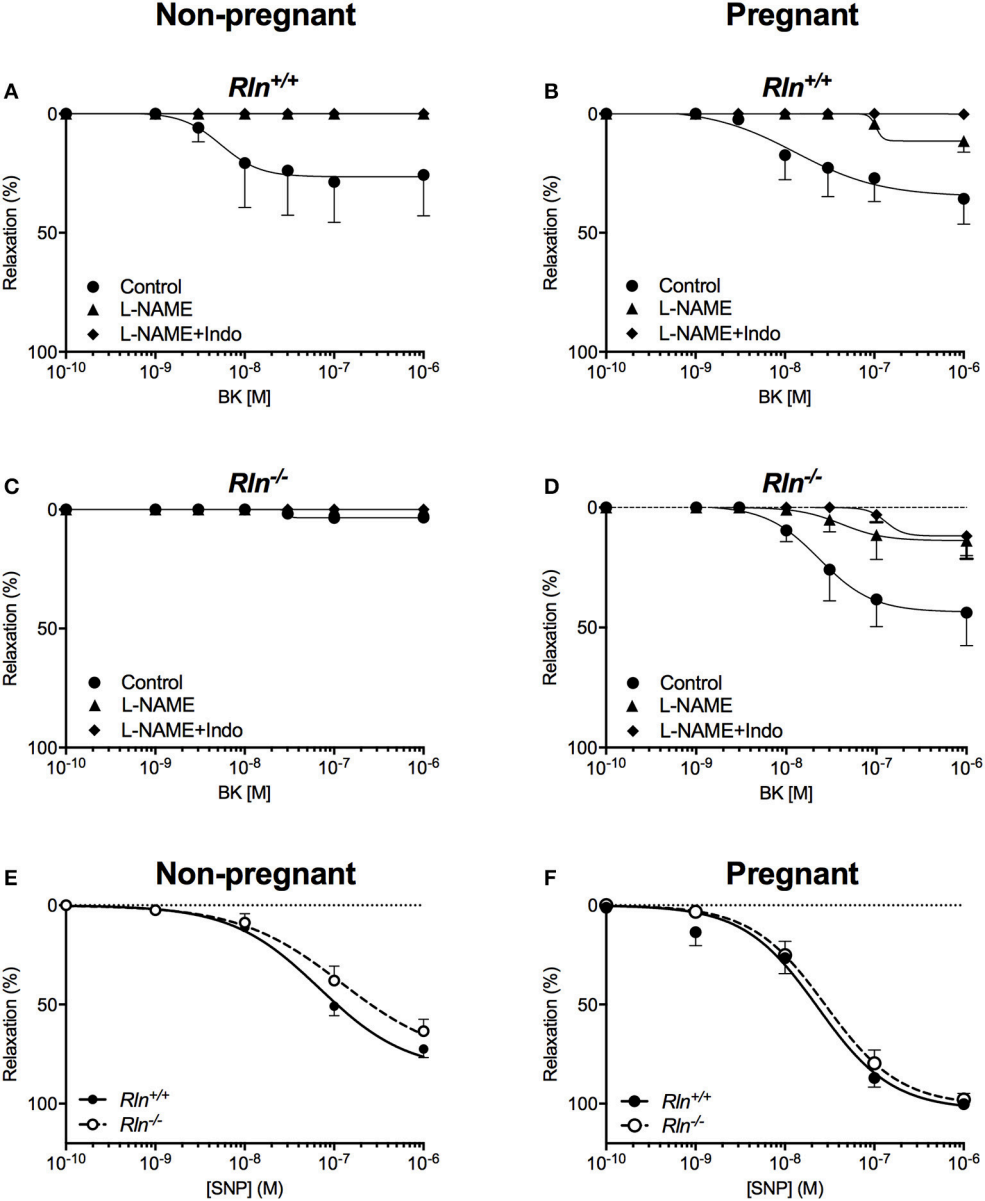
Concentration-response curves to the endothelium-dependent agonist bradykinin (BK) in main uterine arteries from *Rln*^+/+^ **(A)** non-pregnant and **(B)** pregnant mice, and from *Rln*^−/−^ **(C)** non-pregnant and **(D)** pregnant mice after pre-treatment of arteries without (circle; control) or with the NOS inhibitor (triangle) L-NAME or (diamond) L-NAME and the COX inhibitor Indo (L-NAME+Indo). Concentration-response curves to the endothelium-independent agonist sodium nitroprusside (SNP) in main uterine arteries from **(E)** non-pregnant and **(F)** pregnant *Rln*^+/+^ and *Rln*^−/−^ mice (*n* = 7–9 per group).

While ACh evoked maximal endothelium-dependent relaxation, BK evoked a submaximal relaxation, even at the highest concentration used in non-pregnant mice (Figures 5A,C; Table 1). Despite robust BK-mediated relaxation in uterine arteries from non-pregnant *Rln*^+/+^ mice (Figure 5A; Table 1), BK failed to produce significant relaxation in the uterine arteries of non-pregnant *Rln*^−/−^ mice (Figure 5C; Table 1). In contrast to ACh, the relaxation evoked by BK was completely mediated by NO (Figure 5A; Table 1).

In pregnancy, maximal relaxation evoked by BK was not different in uterine arteries from non-pregnant *Rln*^+/+^ mice (Figure 5B; Table 1). However, BK-mediated relaxation was significantly augmented in arteries from the pregnant *Rln*^−/−^ mice (Figure 5D) such that the maximal BK-mediated relaxation was not different to that in arteries from *Rln*^+/+^ mice (Figures 5B,D; Table 1). Most of the BK-mediated relaxation is attributed to NO, however, a small component due to vasodilator prostanoid appeared in pregnancy in uterine arteries from both *Rln*^+/+^ and *Rln*^−/−^ mice (Figures 5B,D; Table 1).

### Endothelium-independent relaxation is intact in the uterine artery of relaxin-deficient mice

There was no significant difference in smooth muscle sensitivity to SNP in uterine arteries of *Rln*^−/−^ and *Rln*^+/+^ mice in non-pregnancy (Figure 5E; Table 1) and pregnancy (Figure 5F; Table 1). In the adaptation to pregnancy, the smooth muscle sensitivity to SNP was unchanged in uterine arteries from *Rln*^+/+^ mice (Figure 5F; Table 1). However, sensitivity to SNP was significantly (*P* = 0.035) increased in *Rln*^−/−^ mice (Figure 5F; Table 1). Maximal relaxation to SNP was significantly increased with pregnancy for uterine arteries from both *Rln*^+/+^ (*P* = 0.0003) and *Rln*^−/−^ (*P* = 0.0012) mice (Figures 5E,F; Table 1).

Sensitivity and maximum response to Iloprost was not different in uterine arteries of *Rln*^+/+^ or *Rln*^−/−^ mice, and were not influenced by pregnancy (Table 1).

### Relaxin deficiency has mild effects on the expression of hypertension-associated genes in late pregnancy

**Figure 6:**
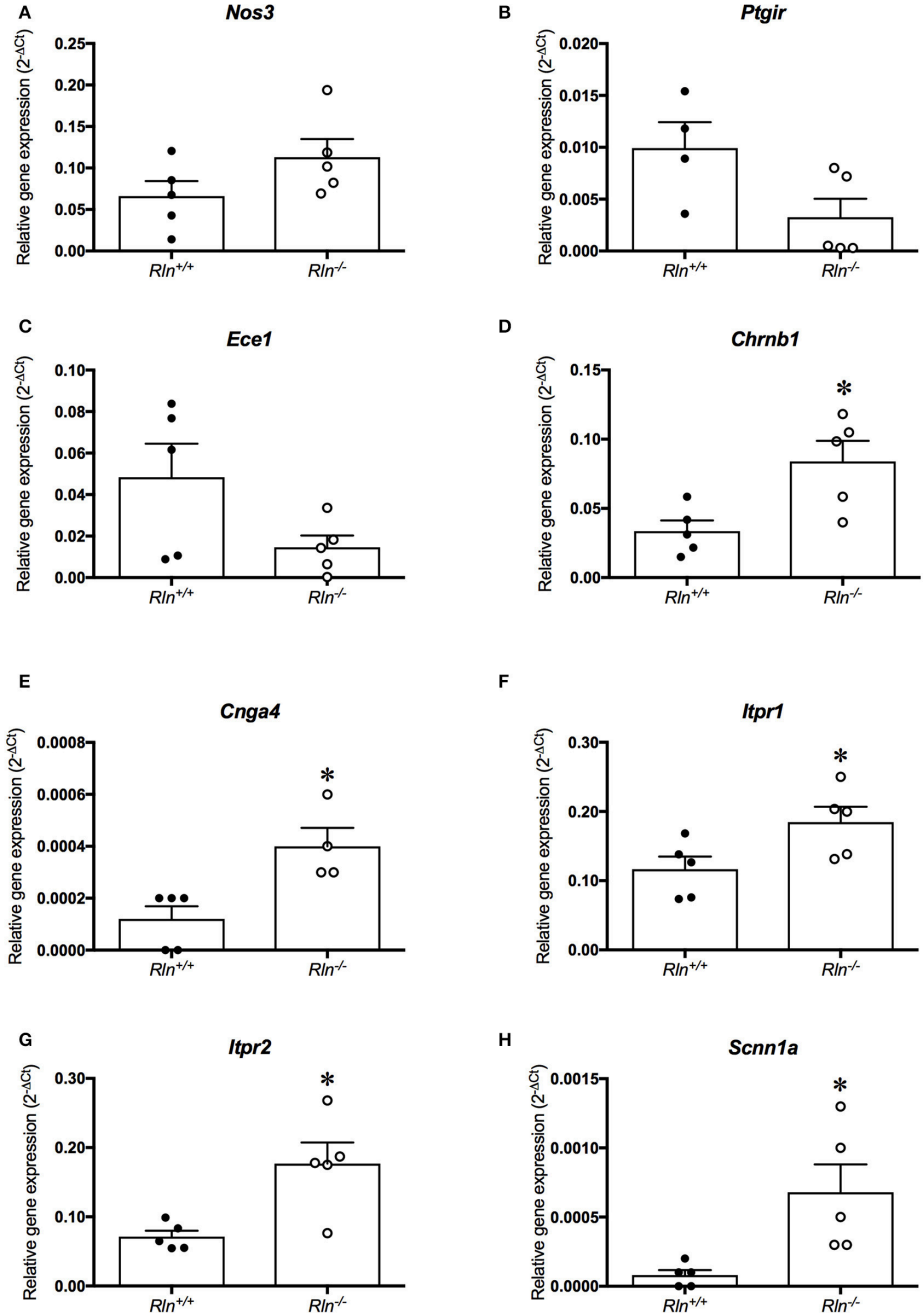
Quantitative PCR analysis of **(A)** endothelial NOS (*Nos3*), **(B)** prostaglandin I_2_ receptor (*Ptgir*), **(C)** endothelin converting enzyme 1 (*Ece1*), **(D)** nicotinic cholinergic receptor type β1 (*Chrnb1*), **(E)** encoding cyclic nucleotide gated channel α4 (*Cnga4*), **(F)** inositol 1,4,5-triphosphate receptor-2 (*Itpr1*), **(G)** inositol 1,4,5-triphosphate receptor-2 (*Itpr2*) and **(H)** sodium non-voltage gated channel type 1α (*Scnn1a*) in the uterine artery of late-pregnant (•) *Rln*^+/+^ and (•) *Rln*^−/−^ mice (*n* = 4–6 per group). ^*^P < 0.05 Student's unpaired *t*-tests based on genotype.

To screen for potential mechanisms regulating the increased myogenic tone and aberrant uterine artery relaxation in late pregnant *Rln*^−/−^ mice, we analyzed 84 genes of interest using a qPCR array. Surprisingly, relaxin deficiency only significantly altered the expression of 8 genes (out of the 84 analyzed). Despite its effects on myogenic tone, relaxin deficiency did not alter genes involved in NO synthesis; endothelial NOS (eNOS; *Nos3*, Figure 6A; Table 2), NOS interacting protein (*Nosip*) or NOS trafficking protein (*Nostrin*; Table 2). Expression of the prostaglandin I_2_ receptor (*Ptgir*) demonstrated a trend toward a reduced expression in *Rln*^−/−^ mice, but failed to reach significance (*P* = 0.06; Figure 6B; Table 2). There was also no significant effect of a relaxin deficiency on the expression of endothelin converting enzyme 1 (*Ece1, P* = 0.08; Figure 6C; Table 2), endothelin-1 (ET-1, *Edn1*), endothelin-2 (*End2*) and the endothelin receptors (ET_A_, *Ednra* and ET_B_, *Ednrb*; Table 2).

**Table 2:** Gene expression in uterine arteries from late pregnant mice.

**Gene**	**Accession number**	**Fold change (⇑or⇓)**	***Rln*^+/+^ raw Ct**		***Rln*^−/−^ raw Ct**		***P*-value (2^−ΔCt^*t*-test)**
			**Mean**	**SEM**	**Mean**	**SEM**	
*Adrb1*	NP_031445	⇑2.31	37.96	1.12	37.29	1.24	0.30
*Agtr1a*	NP_796296	⇓1.27	37.02	1.35	38.11	1.21	0.86
*Agtr1b*	NP_780295	⇓1.48	33.60	0.83	33.86	0.93	0.39
*Atp6ap2*	NP_081715	⇓0.60	30.17	0.53	31.74	0.76	0.01
*Bdkrb1*	NP_031565	⇑1.27	37.55	1.51	37.48	1.12	0.93
*Bmpr2*	NP_031587	⇓0.84	32.12	0.56	33.20	0.83	0.02
*Chrnb1*	NP_033731	⇑2.58	31.58	0.50	31.04	0.47	0.02
*Clic4*	NP_038913	⇓1.44	27.06	0.51	27.35	0.67	0.04
*Cnga4*	NP_001028489	⇑2.03	39.80	0.12	38.01	0.82	0.02
*Ece1*	NP_955011	⇓2.99	31.47	0.87	34.47	1.66	0.08
*Edn1*	NP_034234	⇓1.12	30.30	0.44	30.96	0.71	0.81
*Edn2*	NP_031928	⇑3.34	39.78	1.22	37.62	1.19	0.16
*Ednra*	NP_034462	⇑1.34	33.86	1.59	33.80	1.10	0.26
*Ednrb*	NP_031930	⇓1.88	30.60	0.58	32.34	0.94	0.11
*Hif1a*	NP_034561	⇓0.75	29.43	0.68	30.67	0.76	0.02
*Itpr1*	NP_034715	⇓1.62	29.71	0.52	29.84	0.56	0.04
*Itpr2*	NP_034716	⇑2.37	30.38	0.57	29.96	0.52	0.01
*Nos3*	NP_032739	⇑1.99	30.77	0.84	30.60	0.92	0.13
*Nosip*	NP_079809	⇓1.19	29.47	0.86	30.05	0.59	0.77
*Nostrin*	NP_853525	⇑2.42	37.21	1.15	36.26	0.99	0.22
*Nppc*	NP_035063	⇑2.28	33.13	0.26	32.77	0.22	0.17
*Plcg2*	NP_758489	⇑2.11	32.85	0.92	32.60	0.92	0.10
*Ptgir*	NP_032993	⇓1.13	34.87	1.31	37.07	1.80	0.06
*Ptgs1*	NP_032995	⇓1.25	33.31	0.82	33.81	0.61	0.39
*Ptgs2*	NP_035328	⇓1.04	35.43	1.19	36.80	1.35	0.93

*⇑, Fold change in expression increased based on relaxin deficiency*.

*⇓, Fold change in expression decreased based on relaxin deficiency. Fold change in gene expression, raw Ct-values and P-values for unpaired t-tests (on 2^−ΔCt^; Rln^+/+^ vs. Rln^−/−^) of selected genes of interest from qPCR array on uterine arteries from late-pregnant (d17.5) mice*.

The qPCR assay also revealed novel genes influenced by relaxin deficiency, including nicotinic cholinergic receptor type β1 (*Chrnb1*), encoding cyclic nucleotide gated channel α4 (*Cnga4*), inositol 1,4,5-triphosphate receptor-2 (*Itpr1*) and inositol 1,4,5-triphosphate receptor-2 (*Itpr2*), which were all significantly upregulated by 2-fold or more (*P* < 0.05, Figures 6D–H; Table 2).

### Effects of relaxin deficiency on fetal and placental weights

**Figure 7:**
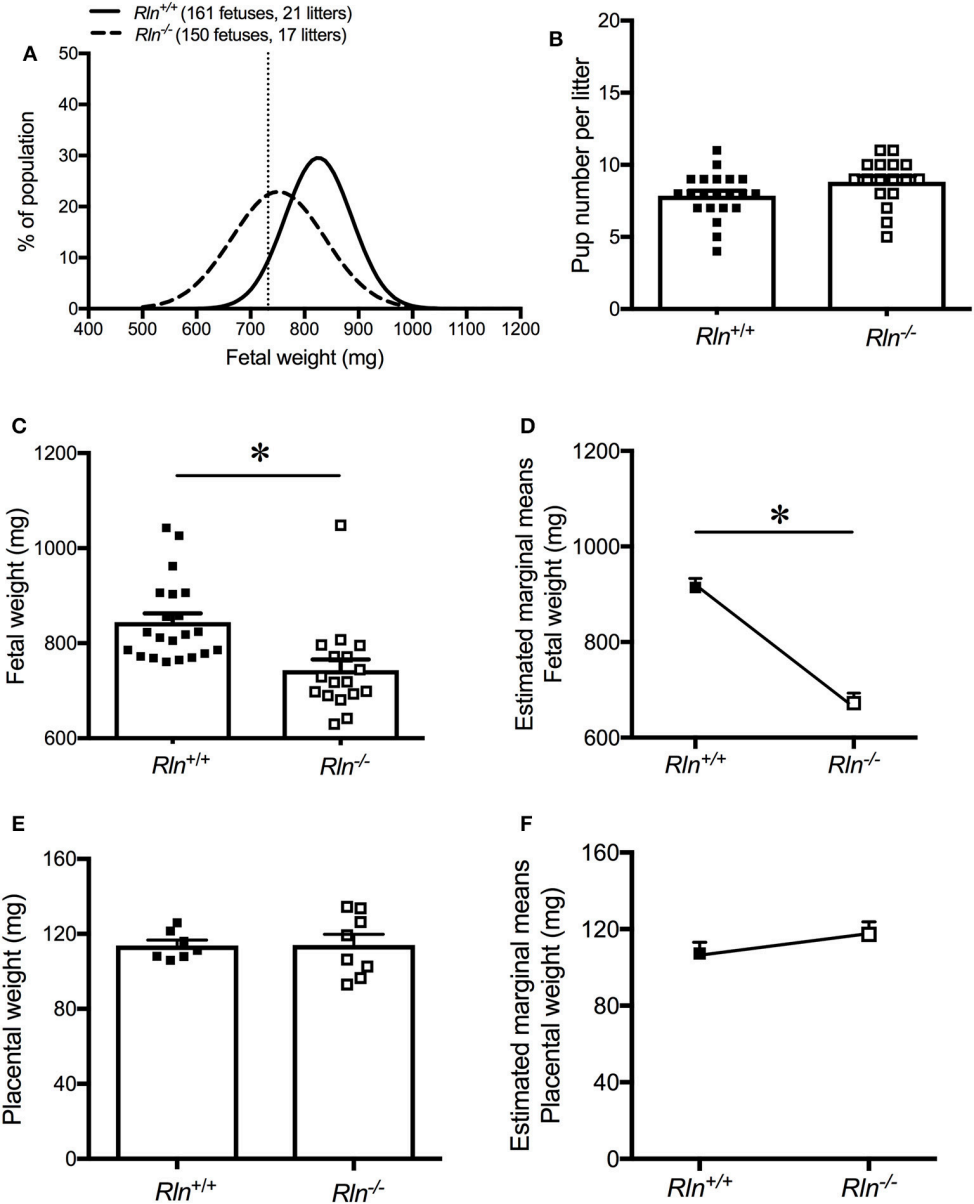
**(A)** Weight distribution of fetuses from day 17.5 pregnant *Rln*^+/+^ and *Rln*^−/−^ mice dams as a % of the study's fetal population. Vertical dashed line represents the 10th centile for fetal weight distribution at 732.4 mg. **(B)** Viable pup number per litter from *Rln*^+/+^ and *Rln*^−/−^ litters. **(C)** Raw average fetal weight (mg), **(D)** estimated marginal means of fetal weight (mg), **(E)** raw average placental weight (mg), and **(F)** estimated marginal means of placental weight (mg) from *Rln*^+/+^ and *Rln*^−/−^ litters. ^*^P < 0.05 Student's unpaired *t*-tests for raw data based on genotype or general linear model for estimated marginal means.

Fetal weight distribution of viable fetuses of *Rln*^−/−^ mice was shifted to the left (indicative of lower weight) with 39% of the fetuses from *Rln*^−/−^ mice having a weight below the 10th centile (<732.4 mg) of the *Rln*^+/+^ mice normal distribution (Figure 7A). The viable number of pups per litter was unchanged based on genotype (*Rln*^+/+^ = 7.9 ± 1.6; *Rln*^−/−^ = 8.8 ± 1.6, Figure 7B). Raw mean weights of litters from pregnant *Rln*^−/−^ mice were significantly reduced relative to *Rln*^+/+^ mice, weighing ~10% less (Figure 7C; Table 3; *P* = 0.001). Once adjusted for dams and litter size, estimated marginal means of fetuses from pregnant *Rln*^−/−^ mice were still significantly reduced relative to *Rln*^+/+^ mice (Figure 7D; Table 3; *P* < 0.001). Interestingly, placental weights at d17.5 of pregnancy were unaffected by genotype, even after adjusting for dams and litter size (Figures 7E,F; Table 3).

**Table 3:** Fetal and placental weights from age-matched *Rln*^+/+^ and *Rln*^−/−^ litters.

	***Rln*^+/+^**	***n***	***Rln*^−/−^**	***n***
Raw fetal weight (mg)	834.7 ± 6.9	21	754.3 ± 7.4^*^	17
Estimated marginal means of fetal weight (mg)	914.2 ± 18.9	21	672.5 ± 20.8^*^	17
Raw placental weight (mg)	113.8 ± 2.9	7	114.0 ± 5.9	8
Estimated marginal means of placental weight (mg)	107.2 ± 5.9	7	117.3 ± 6.5	8

*All values are expressed as mean ± SEM; n, number of litters*.

* *P < 0.05, significantly different compared to Rln^+/+^, Student's unpaired t-tests for raw data or general linear model for estimated marginal means*.

## Discussion

This study revealed that relaxin is a regulator of uterine artery function in non-pregnancy and pregnancy. Importantly, relaxin is required for the normal adaptation of the uterine artery to pregnancy, specifically the reduction in uterine artery myogenic tone. Uterine artery sensitivity to endothelial agonists was significantly reduced in non-pregnant *Rln*^−/−^ mice, with the differences resolved in pregnancy. The underlying contributions of K_Ca_ channels to EDH-mediated relaxation were perturbed in the relaxin deficient mice, a feature that persisted from non-pregnancy to pregnancy. The dysregulation of uterine artery myogenic tone in pregnancy would likely impair uteroplacental perfusion and contribute to the fetal growth restriction in *Rln*^−/−^ mice.

In non-pregnancy, development and regulation of uterine artery myogenic tone was similar between *Rln*^+/+^ and *Rln*^−/−^ mice. Similar findings have also been reported for small renal arteries from non-pregnant *Rln*^−/−^ mice (Novak et al., 2006). In uterine arteries from the non-pregnant animals of both genotypes NO has an important role in the underlying regulation of myogenic tone. There appears to be little role of vasodilator prostanoids as COX inhibition was without significant effect on myogenic tone development. The overall contribution of NO and vasodilator prostanoids to myogenic tone regulation appeared to be similar in arteries from both genotypes.

Suppression of myogenic tone development in the uterine artery during pregnancy has been reported in several species (Veerareddy et al., 2002; Xiao et al., 2010; Hu et al., 2011). In the adaptation to pregnancy we also found that myogenic tone development was significantly reduced in uterine arteries of our late pregnant *Rln*^+/+^ mice. Although NO plays an important role in the regulation of myogenic tone, the role of prostanoids, presumably prostacyclin, was considerably upregulated in uterine arteries of pregnant *Rln*^+/+^ mice. A striking finding in the present study is that the normal suppression of myogenic tone with pregnancy failed to occur in *Rln*^−/−^ mice. The extent of myogenic tone development in uterine arteries from pregnant *Rln*^−/−^ mice was indistinguishable from that in their non-pregnant counterparts. Interestingly, this occurred despite significant upregulation of the role of NO in the regulation of myogenic tone in uterine arteries of pregnant *Rln*^−/−^ mice. Instead, the maintenance of the high level of uterine artery myogenic tone is attributed to the lack of upregulation of the prostanoid component with pregnancy in the setting of relaxin deficiency. These findings are supported by a trend toward decreased prostacyclin receptor mRNA expression. Enzymes involved in the prostaglandin cascade, COX-1 and COX-2, were however unaffected. Further, we have previously demonstrated in pregnant *Rln*^−/−^ mice that there is a deficiency in the production of smooth-muscle derived vasodilator prostanoids in mesenteric arteries (Marshall et al., 2016), which could be restored with a 5 day continuous infusion of relaxin (Marshall et al., 2017a). Thus, reduced production of vasodilator prostanoids and/or expression of their receptors in the uterine artery of pregnant *Rln*^−/−^ mice underpin the maintenance of a high level of myogenic tone.

Relaxin treatment in non-pregnant rats reduces myogenic tone development in renal and mesenteric arteries (Novak et al., 2002), to levels of tone similar to those seen in these arteries during pregnancy (Gandley et al., 2001). This suppression of tone in arteries of non-pregnant rats is attributed to increased bioavailability of NO through increased endothelin and endothelial ET_B_ receptor signaling and increased eNOS activity (Gandley et al., 2001; Novak et al., 2002). However, in our model of relaxin deficiency, a deficit in the role of NO in the regulation of myogenic tone during pregnancy was not observed. In fact, the role of NO was upregulated in uterine arteries of pregnant *Rln*^−/−^ mice. We found no differences in the expression of genes involved in NO synthesis or those associated with the ET_B_ receptor-signaling pathway. Increases in the activity of eNOS and/or bioavailability of NO likely underpin the augmented NO-dependent regulation of myogenic tone in pregnant *Rln*^−/−^ mice and may reflect a compensatory mechanism to counter the failure of the vasodilator prostanoid pathway.

Our results have revealed a key role for endogenous relaxin in mediating the adaptive reduction in myogenic tone of the main uterine artery with pregnancy. This reduction in myogenic tone will facilitate increased perfusion of the uteroplacental unit to support a healthy pregnancy. Indeed, this is supported by similar findings in small renal arteries whereby mid-gestation pregnant rats treated with a relaxin neutralizing antibody (MCA1) no longer demonstrated the normal pregnancy associated decrease in myogenic tone (Novak et al., 2001). Furthermore, MCA1 treated pregnant rats failed to exhibit the gestational increase in cardiac output, global arterial compliance and decrease in systemic vascular resistance that occur during pregnancy in healthy rats (Debrah et al., 2006). Collectively, the current study and previous findings by other groups highlight the importance of relaxin as an integral molecule involved in the adaptation of the maternal cardiovascular system to pregnancy.

Uterine arteries from non-pregnant *Rln*^−/−^ mice exhibited endothelial vasodilator dysfunction when stimulated with ACh or BK. ACh released the complement of vasodilators including NO, EDH and a vasodilator prostanoid likely prostacyclin. Sensitivity to ACh was significantly reduced in the *Rln*^−/−^ mice, although maximal relaxation was not different between genotypes. Similarly, in a study of male *Rln*^−/−^ mice, mesenteric arteries had reduced sensitivity to ACh-mediated relaxation (Leo et al., 2014a). The impairment in the males is attributed to the upregulation of the prostanoid vasoconstrictor pathway, however, we have no evidence that the impairment in *Rln*^−/−^ females is due to this. Our study also correlates with relaxin treatment studies (Leo et al., 2014b, 2017; Ng et al., 2016). Following blockade of NO synthesis, there was no difference in sensitivity to ACh between genotypes, however once prostanoid production was inhibited, the sensitivity to ACh and thus EDH-mediated relaxation was impaired. Further investigation of the role of the K_Ca_ channels involved in EDH-mediated relaxation revealed that the contribution of SK_Ca_ channels was diminished in uterine arteries of the non-pregnant *Rln*^−/−^ mice. Impairment of SK_Ca_ channel expression and/or activity in arteries has been documented in a variety of cardiovascular diseases (Félétou, 2016).

In uterine arteries the overall endothelium-dependent relaxation evoked by BK was only a fraction of that evoked by ACh. Whereas ACh evoked the release of a variety of vasodilators from the endothelium of uterine arteries of mice, as previously published (Cooke and Davidge, 2003), BK evoked a relaxation that was entirely mediated by NO in non-pregnant mice. Interestingly, the BK-mediated relaxation was virtually non-existent in arteries from non-pregnant *Rln*^−/−^ mice. The absence of a response to BK was likely also exacerbated by the impaired uterine artery smooth muscle sensitivity to NO in non-pregnant *Rln*^−/−^ mice. In male rats, relaxin treatment (an acute intravenous injection or 72 h continuous infusion) selectively enhanced BK-mediated relaxation in the mesenteric artery by upregulating prostacyclin production (Leo et al., 2014b, 2016b), while a 48 h relaxin infusion enhanced BK-mediated relaxation via the NO pathway (Leo et al., 2016b). Relaxin is hypothesized to signal thorough heterodimers composed of the relaxin receptor and BK receptors (RXFP1-B_2_R) to influence dilation (Leo et al., 2016a). Due to tissue limitations of the uterine artery of non-pregnant mice, we were unable to further investigate whether a relaxin deficiency has influenced these heterodimers or genes associated with ACh-mediated relaxation.

The differences in ACh sensitivity and BK responsiveness observed between uterine arteries of *Rln*^+/+^ and *Rln*^−/−^ mice were resolved with pregnancy. Endothelial vasodilator function is upregulated in pregnancy, with increased activity of the NO, prostacyclin and EDH pathways under the influence of steroid hormones such as estrogen (Weiner et al., 1994; Vagnoni et al., 1998; Zhang et al., 2001; Egan et al., 2004; Booth et al., 2008; Gokina et al., 2010). Even though ACh sensitivity for EDH-mediated relaxation was not different in pregnancy between genotypes, there remained underlying dysregulation of IK_Ca_ and SK_Ca_ channel contributions in arteries from the relaxin deficient mice. The role of IK_Ca_ channels was upregulated while that of SK_Ca_ channels was reduced in uterine arteries of *Rln*^−/−^ mice. Relaxin has been previously demonstrated to stimulate myometrial calcium-activated potassium channel activity (Meera et al., 1995), and upregulate IK_Ca_ channel activity in rat mesenteric arteries and cerebral parenchymal arterioles (Leo et al., 2016a). However, relaxin's potential effects on these calcium-activated potassium channels during pregnancy in the uterine artery remain unexplored. Whether the disparity in IK_Ca_ and SK_Ca_ channel contributions between *Rln*^−/−^ and *Rln*^+/+^ mice are due to differences in channel expression or signaling mechanisms (Gokina et al., 2010; Félétou, 2016) will need to be explored in future studies.

To maximize the experimental output of the limited tissue available a qPCR array analysis approach assessed how relaxin deficiency affected the expression of 84 different genes in main uterine artery of late pregnant mice (Moradipoor et al., 2016). This enabled a broad screen of genes associated with the cardiovascular system, which could not be achieved through other quantitative methods. That said, we did observe trends in gene expression that did not reach significance, and these analyses would have benefitted from additional tissue if it was available. Of the 8 genes significantly modulated by relaxin deficiency, the majority were novel and unexpected. Relaxin deficiency had no significant effect on the genes involved in NO or PGI_2_ synthesis, but upregulated the mRNA expression of the ACh receptor *Chrnb1* (Albuquerque et al., 2009). As the uterine arteries from *Rln*^−/−^ mice no longer demonstrated a reduced sensitivity to ACh during late pregnancy in the absence of inhibitors, this may be due to an increase in ACh receptor expression. Of the novel genes influenced by relaxin deficiency *Cnga3*, a cyclic nucleotide gated channel subunit, is involved in signal transduction and can be influenced by calcium ions (Dai et al., 2014), making it an interesting find. The hypoxia-inducing factor α (*Hif1*α), a subunit of a hypoxia acting transcription factor (Benita et al., 2009) was downregulated in the uterine artery of late pregnant *Rln*^−/−^ mice. Whether and to what extent these changes in gene expression translate to the altered phenotype of the main uterine artery remain to be elucidated.

In summary, endogenous relaxin has an obligatory role in the normal pregnancy-associated suppression of myogenic tone in the uterine artery. In pregnancy endogenous relaxin critically upregulates the vasodilator prostanoid-mediated modulation of myogenic tone to support uteroplacental perfusion and fetal growth.

## Author contributions

LP and MT designed the research project. SM, SS, MJ, KO, and MT conducted the research. SM bred mice, screened for estrus, established pregnancy, monitored all mice and completed all tissue collection. SM, MT, and SS wrote the manuscript. SM, SS, MJ, and MT analyzed the data and performed the statistical analyses. All authors read and approved the final manuscript.

### Conflict of interest statement

The authors disclose that LP was a paid consultant for Novartis Pharma AG from 2012 to 2015. The other authors declare that the research was conducted in the absence of any commercial or financial relationships that could be construed as a potential conflict of interest.
